# Associations of Bone Mineral Density and Lead Levels in Blood, Tibia, and Patella in Urban-Dwelling Women

**DOI:** 10.1289/ehp.10977

**Published:** 2008-02-26

**Authors:** Keson Theppeang, Thomas A. Glass, Karen Bandeen-Roche, Andrew C. Todd, Charles A. Rohde, Jonathan M. Links, Brian S. Schwartz

**Affiliations:** 1 Department of Environmental Health Sciences; 2 Department of Biostatistics, and; 3 Department of Epidemiology, Bloomberg School of Public Health, Johns Hopkins University, Baltimore, Maryland, USA; 4 Department of Community and Preventive Medicine, Mount Sinai School of Medicine, New York, New York; 5 Department of Medicine, Johns Hopkins School of Medicine, Baltimore, Maryland, USA

**Keywords:** biomarkers, bone mineral density, dual energy X-ray absorptiometry, epidemiology, lead, X-ray fluorescence, women

## Abstract

**Objective:**

The objective of this study was to evaluate the relations between bone mineral density (BMD) and lead in blood, tibia, and patella and to investigate how BMD modifies these lead biomarkers in older women.

**Design:**

In this study, we used cross-sectional analysis.

**Participants:**

We studied 112 women, 50–70 years of age, including both whites and African Americans, residing in Baltimore, Maryland.

**Measurements:**

We measured lumbar spine BMD, blood and bone lead by dual energy X-ray absorptiometry, anodic stripping voltammetry, and ^109^Cd-induced K-shell X-ray fluorescence, respectively. We measured vitamin D receptor and apolipoprotein E (*APOE*) genotypes using standard methods.

**Results:**

Mean (± SD) BMD and lead levels in blood, tibia, and patella were 1.02 ± 0.16 g/cm^2^, 3.3 ± 2.2 μg/dL, 19.7 ± 13.2 μg/g, and 5.7 ± 15.3 μg/g, respectively. In adjusted analysis, higher BMD was associated with higher tibia lead levels (*p* = 0.03). BMD was not associated with lead levels in blood or patella. There was evidence of significant effect modification by BMD on relations of physical activity with blood lead levels and by *APOE* genotype on relations of BMD with tibia lead levels. There was no evidence that BMD modified relations between tibia lead or patella lead and blood lead levels.

**Conclusions:**

We believe that BMD represents the capacity of bone that can store lead, by substitution for calcium, and thus the findings may have relevance for effect-size estimates in persons with higher BMD.

**Relevance to clinical practice:**

The results have implications for changes in lead kinetics with aging, and thus the related risk of health effects associated with substantial early- and midlife lead exposure in older persons.

Skeletal lead represents approximately 90–95% of an adult’s current body burden of lead (Barry 1975; Wittmers et al. 1988); because of lead’s decades-long residence time in tibia, measurement of tibia lead is an estimate of lifetime cumulative lead dose. Bone may release its lead content into the bloodstream in the course of normal bone metabolism and may release lead at increased rates during active bone demineralization in later life (Gerhardsson et al. 1993; Hernandez-Avila et al. 2000; Rosen 1983; Silbergeld et al. 1988; Symanski and Hertz-Picciotto 1995). The release of lead from bone with aging is of particular concern to older women in the United States because of high and long-term past exposures, particularly before the 1980s. Women also experience more rapid bone mineral loss than men beginning in the fourth decade of life, which can result in loss of up to 50% of bone mineral density (BMD) by 80 years of age (Ferguson and Steffen 2003).

After menopause, the rate of bone loss increases up to fourfold on average, from 0.5 to 1% annually before menopause to 2–3% annually after menopause (World Health Organization 1994). These rates are influenced by race/ethnicity [faster rates in whites (Berglund et al. 2000; Marcus et al. 1996; Symanski and Hertz-Picciotto 1995)], genetic factors, hormones, nutrition, physical activity, body weight, and medications (Marcus et al. 1996; Zmuda et al. 1999). Studies have raised concerns that changes in bone turnover rates that occur with aging may also influence the kinetics of lead in bone and blood (Fullmer 1992; Nash et al. 2004; Smith et al. 1995). This could result in greater release of lead from bone, higher blood lead levels, and increased deposition in critical target organs, thus increasing the risk of lead-related health outcomes. This raises questions about what factors may modify the risk associated with the early- and midlife legacy of high lead exposure experienced by the current population of older Americans (Bellinger 2000).

Lead in bone probably causes important bone toxicity (Hicks et al. 1996; Klein and Wiren 1993; Pounds et al. 1991; Ronis et al. 2001). Previous studies suggest that lead is associated with decreases in the function of osteoblasts, which are responsible for bone formation (Hass et al. 1967; Long et al. 1990a, 1990b). In addition, two cross-sectional studies in lead workers reported an association of lead exposure with increases in circulating levels of parathyroid hormone (PTH) and 1,25-dihydroxyvitamin D_3_, which regulate the levels of serum calcium and bone metabolism (Kristal-Boneh et al. 1998; Mason et al. 1990). Although evidence suggests that lead may be associated with lower calcium content in bone and impaired skeletal development (World Health Organization 1994), the associations between BMD, a measure that summarizes aspects of the general health of bone and is used to predict the risk for osteoporotic fractures, and lead in bone and blood have not been well characterized. A study in rats (Gruber et al. 1997) found that lead exposure was associated with lower BMD, whereas another study (Escribano et al. 1997) reported lead exposure associated with higher BMD.

In humans, studies have reported both positive associations between BMD and blood lead levels in children (i.e., the higher the BMD, the higher the blood lead level) (Campbell et al. 2004) and negative ones in adults (Campbell and Auinger 2007; Nash et al. 2004), but these results have not allowed inferences about the likely causal direction of this relation. Only one study of 73 selected, female, highly exposed former smelter workers evaluated the association between BMD and tibia lead (Potula et al. 2006); this study found that spine BMD decreased with increasing blood lead over time but found no association between BMD and tibia lead levels. To date, no studies have evaluated the association of BMD with levels of lead in patella, a trabecular and therefore possibly more metabolically active bone site. Finally, no studies have evaluated potential effect modification by BMD on the relations between blood and bone lead levels, an analysis directly relevant to hypotheses regarding BMD and release of lead from bone.

A growing literature suggests that genetic polymorphisms may modify the toxicokinetics of lead in bone and blood (Chuang et al. 2000; Schwartz et al. 2000; Wetmur et al. 1991). Two genes thought to be relevant to deposition or release of lead or calcium from bone are the vitamin D receptor (*VDR*) and apolipoprotein E (*APOE*) (Cooper and Umbach 1996; Peacock 1995; Stulc et al. 2000). Previous studies suggest that the *VDR Bsm*1 restriction site *BB* genotype, the *VDR Fok*1 restriction site *ff* genotype, and the *APOE* ε4 allele are associated with low BMD and higher rates of bone mineral loss (Fleet et al. 1995; Shiraki et al. 1997; Zajickova et al. 2002; Zmuda et al. 1999). In addition, studies have found that subjects with the *BB* genotype have higher bone lead, chelatable lead, and blood lead levels than subjects with the *bb* genotype (Chuang et al. 2000; Schwartz et al. 2000). To date, no studies have evaluated relations of bone lead concentrations with *VDR Fok*1 and *APOE* genotypes, nor whether *VDR Bsm*1, *Fok*1, and *APOE* genotypes modify the relations of bone lead concentrations with BMD.

In this study, we evaluated associations between BMD and lead in blood, tibia, and patella, as well as effect modification by BMD on relations among lead in these three pools, in community-dwelling urban women in Baltimore, Maryland, 50–70 years of age with diversity by race/ethnicity. We also evaluated effect modification of these relations by the two polymorphic genes.

## Materials and Methods

### Study population and design

Study participants represented a subsample of women who completed the third visit (from September 2004 through May 2005) of the longitudinal Baltimore Memory Study. Study population, selection, and recruitment for this study have been previously reported (Martin et al. 2006; Schwartz et al. 2004; Shih et al. 2006). Women with complete information on lead biomarkers and the relevant genotypes were randomly selected by strata of race/ethnicity and *VDR* genotype (by the *Fok*1 restriction enzyme) to ensure that we had approximately similar numbers of subjects in each race/ ethnicity and *VDR Fok*1 genotype category. Selected subjects were then contacted by phone to ascertain their interest in participating in this substudy. We telephoned each subject until we had established a disposition after a maximum of 10 attempts. A total of 290 women were contacted, and 112 agreed to participate and completed BMD measurement, a sample size based on a balance of statistical power and budgetary considerations; 42 had BMD measurement at the Johns Hopkins Outpatient Center and 70 at the Johns Hopkins Bayview Medical Center. There were no differences in levels of lead in blood, tibia, or patella, age, the physical activity measures, menopausal status, or race/ethnicity between those who were contacted and did (*n* = 112) or did not (*n* = 178) participate for the BMD substudy (all *p*-values > 0.05). All participants provided written informed consent at the beginning of the visit and were paid $25 for their participation in the BMD substudy. The Committee for Human Research at the Johns Hopkins Bloomberg School of Public Health reviewed and approved the study.

### Data collection

Data collection methods have been previously reported (Martin et al. 2006; Schwartz et al. 2004; Shih et al. 2006). In brief, all subjects were scheduled for three visits at approximately 14-month intervals; 1,140 completed the first visit (V1), between 30 May 2001 and 20 September 2002; 1,022 (89.6%) completed the second visit (V2), between 1 October 2002 and 31 December 2003; and 943 (82.7%) completed V3, between 1 January 2004 and 31 March 2005. Of the 197 who did not return for V3, 101 (9% of those enrolled) refused, 23 (2%) were too ill, 21 (2%) were deceased, 38 (3%) were lost to follow-up, and 14 (1%) moved out of state. However, in comparing the 943 subjects who completed V3 with the 77 who completed V2 but not V3 and the 197 who completed V1 but not V3, there was no evidence of selective dropout by age, sex, race/ethnicity, education, or wealth (all *p*-values by chi-square or analysis of variance > 0.05). At each study visit, data were collected in the following order: neurobehavioral testing, blood pressure, height, weight, spot urine collection, structured interview, and a 10-mL blood specimen by venipuncture. Lead in bone was measured by X-ray fluorescence (XRF) during the structured interview.

We used data from all three visits in the cross-sectional analysis reported here. Data obtained from the first visit included demographics, self-reported menopausal status, medications (including hormone replacement therapy), smoking history, alcohol consumption, blood lead levels, and genotypes. Data obtained from the second visit included tibia lead levels, dietary intake using the Block 98.2 Dietary Questionnaire (Berkeley Nutrition Services, Berkeley, CA), and physical activity using the Yale Physical Activity Survey (YPAS), a valid and reliable 43-item self-report instrument designed for epidemiologic studies of older adults (Dipietro et al. 1993; Young et al. 2001). We used two indices in the analysis, the total energy expenditure index (termed “Yale energy index”) and the vigorous activity index (termed “Yale vigorous index”). Data obtained at the third study visit were patella lead levels.

### Laboratory methods

We measured BMD at the lumbar spine (L1–L4) by the dual energy X-ray absorptiometry (DEXA) technique. We used a Hologic QDR-4500A elite fan beam bone densitometer with a motorized table and C-arm (Hologic Inc., Bedford, MA) located at the Division of Nuclear Medicine, Department of Radiology, at the Johns Hopkins Outpatient Center, and at the Beacham Osteoporosis Center at the Johns Hopkins Bayview Medical Center. Subjects were placed in the supine position on a table and scanned in the antero-posterior projection with a 15-sec measurement. The accuracy error of assessing BMD by DEXA is < 5%, and the precision error is < 1% *in vivo* (Christenson 1997; Patel et al. 2000). BMD was measured in grams per square centimeter and was expressed as a *z*-score (the number of standard deviations above or below the age-matched reference value) and a *t*-score (the number of standard deviations above or below the young reference value, the mean BMD for healthy 30-year-old women from the reference data from the BMD laboratories).

We measured blood lead with anodic stripping voltammetry (in micrograms per deciliter) as previously reported (Schwartz et al. 2004). As an index of reliability, the coefficients of variability (CV) for 5.9 μg/dL of blood lead were 11% (intraday CV) and 7% (interday CV). The limit of detection was 1 μg/dL. Tibia and patella lead were measured in units of micrograms lead per gram bone mineral, via a 30-min measure at the mid-tibia shaft and left-center patella, respectively, using ^109^Cd-based K-shell XRF (Todd 2000a, 2000b; Todd and McNeill 1993; Todd et al. 2002). Studies have demonstrated that bone lead measurement by XRF is a valid (Somervaille et al. 1986; Todd et al. 2002) and reliable (Muntner et al. 2007; Todd et al. 2000, 2001) technique. We performed genotyping in the laboratory of the Malaria Institute in the Johns Hopkins Bloomberg School of Public Health. We measured polymorphisms in two genes thought relevant to blood or bone lead levels, *APOE* and *VDR* (the latter using two restriction enzymes, *Bsm*1 and *Fok*1), by previously reported methods (Audi et al. 1999; Chuang et al. 2000; Schafer et al. 2005; Stewart et al. 2002; Zmuda et al. 1999).

### Statistical analysis

The primary goals of this cross-sectional analysis were as follows: First, we aimed to evaluate associations of the three lead biomarkers with BMD to understand how lead in bone and blood may influence BMD, and vice versa. Because there are strong biologic rationales for evaluating the directionality of the relations between BMD and tibia lead in both directions (e.g., lead in bone is toxic to bone cells, thus influencing BMD, whereas bone mineral is lost with aging, thus influencing the denominator in the bone lead concentration measured by XRF), we created models to do so. Although the analysis was cross-sectional, we believe that hypotheses can be generated about the likely causal direction because of the different timing of lead biomarker and BMD measurement and the fact that deposition of lead in bone is likely to reflect a much longer time period than are changes in BMD. Second, we aimed to examine whether these relations were modified by the *APOE* and *VDR* polymorphisms. Finally, we aimed to examine effect modification by BMD on the relations of important predictor variables thought to be relevant to bone mineral and bone lead kinetics (e.g., age, sex, race/ethnicity, physical activity, dietary intake, physical activity, genotypes) with the three lead biomarkers.

Because of departures from the normality assumption, blood lead was natural logarithm (ln) transformed before regressing on covariates; the adequacy of this transformation was confirmed by examination of the distributions of the residuals of the final regression models. In the presentation of results, we back-transformed regression coefficients from the analysis of ln-transformed concentrations to facilitate interpretation of results in the original blood lead measurement scale. The resulting coefficients estimate ratios of median concentrations comparing across predictor levels.

Because XRF measurement of bone lead concentration can, due to measurement uncertainty, produce negative point estimates when the true bone lead concentration is close to zero, in our analysis, we kept all point estimates (including negative values) of bone lead concentrations, which yields less bias and more efficient comparisons (Kim et al. 1995). Negative values were particularly common for patella lead (37.5% had values that were less than or equal to zero). We thus used TOBIT regression (Austin et al. 2000; Pindyck and Rubinfeld 1998) with left truncation at 0 μg/g to model tibia and patella lead. In brief, TOBIT is used to model data whose distribution is limited compared to the normal distribution (e.g., distribution is truncated or censored). In the TOBIT model, it is assumed that there is an underlying latent variable of interest generated by the linear regression model with an error term that is normally distributed, *y_i_* = *X_i_*β= ε_i_, where ε_i_ ~ *N*(0, σ^2^), but for which we have only observed *y_i_* = max(0,*y_i_*). The TOBIT model uses maximum likelihood for estimation. To evaluate whether our results were sensitive to the modeling method, we compared the results from TOBIT models to those from multiple linear regression. The associations and our conclusions were similar (data not shown).

We used statistical software programs of the STATA Corporation (version 9; StataCorp, College Station, TX). To describe differences in lead levels and selected subject characteristics by BMD levels and to model effect modification by BMD on the relations between lead biomarkers and their predictors, we dichotomized BMD into high and low groups at a BMD *t*-score of zero. We first performed univariate analyses by BMD group to compare lead biomarkers, subject characteristics, and covariates in women with high and low BMD. We used *t*-tests and analysis of variance to evaluate the statistical significance of the differences of mean lead biomarkers and covariates by BMD group and genotypes. We used multiple linear regression to control for covariates and evaluate potential confounding on the associations between BMD and the three lead biomarkers.

Covariates that were examined included weight, height, body mass index (BMI, in kilograms per square meter), tobacco and alcohol consumption, medications [e.g., oral cortico-steroids, hormone replacement therapy (HRT)], and lifestyle and other risk factors for bone mineral loss (e.g., specific medical conditions, dietary history, physical activity, age, sex, race/ethnicity). A variable was retained in the final models if it was *a*) known to be important based on prior studies, *b*) a significant predictor (*p* < 0.05) of lead biomarkers, *c*) a confounder (based on a 10% change in regression coefficients), or *d*) an effect modifier of the relations of interest. The final regression model was used for exploratory analysis of effect modification, by inclusion of cross-product terms for testing of specific effect modification hypotheses, one at a time.

We performed regression diagnostics, including examination of distributions, residuals, partial residual plots, and variance inflation factors, to evaluate the assumptions of linear regression (e.g., normality of residuals, linearity, homoscedasticity, independence). We also evaluated potential nonlinearity by inclusion of quadratic terms in the linear regression models (retained if *p* < 0.05).

## Results

### Description of study subjects

Of the 112 female participants, 42% were of African-American race/ethnicity. The average age (± SD) of the participants was 59.7 ± 5.7 years at enrollment; 83% were postmenopausal, 14% pre-menopausal, and 3% suspected they were perimenopausal. The stratified random selection of study subjects by *VDR Fok*1 genotype was generally successful, as subjects consisted of 41 (36.6%), 38 (33.9%), and 33 (29.5%) of the *VDR Fok*1, *FF*, *Ff*, and *ff* genotypes, respectively. There were no differences (all *p*-values > 0.05) in levels of lead in blood, tibia, or patella, or in age, physical activity measures, race/ethnicity, hormone replacement therapy use, menopausal status, or *APOE* genotype among women who did and did not participate in the BMD substudy (Table 1).

The mean BMD of the lumbar spine and blood, tibia, and patella lead levels were 1.03 ± 0.16 g/cm^2^, 3.3 ± 2.2 μg/dL, 19.7 ± 13.2 μg/g, and 5.7 ± 15.3 μg/g, respectively. There was no difference in mean BMD levels between the testing site of measurements [*p* > 0.05 for comparing measurements at the Johns Hopkins Outpatient Center (mean = 1.034 ± 0.025 g/cm^2^) with those at the Johns Hopkins Bayview Medical Center (mean = 1.027 ± 0.018 g/cm^2^)]. The prevalences of the *APOE* ε4 allele and the *VDR Bsm*1 *BB* genotype were 26.8% and 11.6%, respectively. There were differences by BMD group in the mean of the Yale energy index, and in the prevalence of the *APOE* ε4 allele and the use of HRT (all *p* < 0.05) (Table 1). Even though there was a higher proportion of subjects in the high BMD group who reported using HRT than in low BMD group (Table 1), the difference in the mean BMD by HRT use did not achieve statistical significance.

### Modeling BMD: associations of lead biomarkers with BMD and effect modification by polymorphic genes on these relations

In crude analysis, blood, tibia, and patella lead levels were not associated with BMD levels (*p* > 0.10). However, in the adjusted analysis of BMD levels, higher tibia lead was associated with higher BMD levels, whereas the *APOE* ε4 allele was associated with lower BMD levels (Table 2, model 1). Adjusting for weight and *APOE* genotype, the average BMD was 0.002 g/cm^2^ higher for every 1.0 μg/g increase in tibia lead (*p* = 0.04) and 0.057 g/cm^2^ lower for subjects with the ε4 allele than subjects without the allele (*p* = 0.05) (Table 2, model 1). Menopausal status was not associated with BMD. We next examined effect modification by potential moderators on the relations of tibia lead levels with BMD. We found that *APOE* genotype modified the relations of tibia lead with BMD (Table 2, model 2). On average, the change in the adjusted mean BMD per 1 μg/g change in tibia lead levels was not the same for women with and without the *APOE* ε4. For women without the allele, the mean BMD increased 0.004 g/cm^2^ per 1 μg/g increase in tibia lead levels, whereas the mean BMD decreased 0.001 g/cm^2^ per 1 μg/g increase in tibia lead levels in women with the allele (*p* < 0.01). We did not observe any associations of *VDR* genotypes with BMD levels or effect modification by *VDR* genotypes on relations between the three lead biomarkers and BMD.

### Modeling lead biomarkers: associations of BMD with lead biomarkers and effect modification by BMD on the relations of predictor variables with lead biomarkers

BMD was not associated with blood lead levels in either crude or adjusted analyses (Table 3). However, BMD modified the relation of the Yale energy index with blood lead levels (Table 3, model 1; for these models, BMD was divided into high and low groups for ease of interpretation). The adjusted median blood lead in those with low BMD was 0.3% higher per 100 kcal/week increase in the Yale energy index, whereas the median blood lead was 0.3% lower per 100 kcal/week increase in women with a high BMD (*p* < 0.01). There was no evidence of effect modification by BMD on the relations of patella lead or tibia lead with blood lead levels (Table 3, models 2 and 3, respectively). Menopausal status was not associated with the lead biomarkers, nor did it modify relations of BMD with lead biomarkers (but there was little variation in menopausal status because 85% of subjects were postmenopausal).

With tibia lead as the dependent variable, BMD was not associated with levels in the crude analysis. However, in the adjusted analysis, BMD was positively associated with tibia lead levels (*p* = 0.03); tibia lead levels were 0.01 μg/g higher for every 1 mg/cm^2^ increase in BMD (Table 4, model 1). Both *APOE* genotype and the Yale energy index modified the relation of BMD with tibia lead (Table 4, models 2 and 3, respectively). The *APOE* genotype differences in adjusted mean tibia lead were different between women with high BMD and low BMD (*p* < 0.01). In the low BMD group, women with the ε4 allele had tibia lead levels that were 6.2 μg/g higher compared with those without the allele, but in the high BMD group, women with the allele had tibia lead levels that were 9.9 μg/g lower compared with those without the allele (Table 4, model 2). Finally, for those in the low BMD group, adjusted tibia lead levels were 0.09 μg/g higher per 100 kcal/week increase in the Yale energy index. In contrast, for those in the high BMD group, mean tibia lead levels were 0.01 μg/g lower per 100 kcal/week increase in the index (*p* = 0.03, Table 4, model 3).

With patella lead as the dependent variable, we did not observe associations with BMD in either crude or adjusted analyses (data not shown). There was also no evidence that BMD modified relations of important predictor variables with patella lead levels (data not shown).

## Discussion

In an earlier report that included the entire sample of > 900 adults in the Baltimore Memory Study who completed both tibia and patella lead measurements (Theppeang et al., in press), we made several observations that motivated us to measure BMD in a sub-sample of women. For example, we found that African Americans had significantly higher tibia lead levels than did whites (on average, almost 30% higher) (Theppeang et al., in press), but no difference in patella lead levels. Higher tibia and patella lead levels were both associated with increasing age. Use of HRT and greater physical activity were both independently associated with lower blood lead levels, probably because there was less release of lead from bone with demineralization (Webber et al. 1995). *APOE* genotype modified associations of sex with patella lead levels. Finally, women had significantly lower blood and patella lead levels than did men. As many of these associations could be explained by the kinetics of lead in bone or bone mineral, further investigation required that we measure BMD. Because bone demineralization with increasing age is much greater in women, we decided to optimize the study design by limiting BMD measurement to a random sample of 112 women, stratified by selected genotypes and race/ethnicity. Although there were interesting race/ethnic differences in analysis of the complete sample, we found no consistent race/ethnic associations in the BMD subsample. Our ability to evaluate whether race/ethnicity modified relations of menopausal status with BMD or lead biomarkers was limited by the limited variation in menopausal status. We found no evidence that race/ethnicity modified relations of age, physical activity, or other predictor variables with lead biomarkers or BMD.

In the adjusted analysis, we found that higher tibia lead levels were associated with higher BMD. However, BMD was measured at the lumbar spine (L1–L4), which consists of more than 66% trabecular bone (Einhorn 1992; Riggs et al. 1982; World Health Organization 1994), whereas lead in bone was measured in both tibia and patella. Tibia is approximately 99% cortical bone (Giangregorio and Webber 2004), and patella is approximately 99% trabecular bone (Hughes et al. 1998). These large differences in bone type by the three sites of the measurements thus complicate interpretation of the associations. It may also be inaccurate to assume that the “mineral” of BMD measurements is identical to the “mass of bone mineral” denominator of a bone lead XRF result (which is actually a matrix conversion factor of the ratio of the elastic scattering cross-sections for calcium hydroxyapatite and calcium sulfate dihydrate, applied to the mass of calcium dihydrate). This contrast is also evident in the differences in the units of the two measures (BMD is mass per unit area, whereas XRF samples a volume of bone).

We considered three other potential explanations for the association of BMD with tibia lead. First, lead in bone may interfere with BMD measurement, resulting in spuriously high BMD estimates. Second, lead may be toxic to osteoblasts, osteoclasts, or both, influencing the biology of bone and thus mineral deposition and mobilization. Finally, bone with higher mineral content may have more binding and deposition sites for lead, so bone with higher density simply allows more deposition sites for lead.

Concerning the first explanation, there is some direct evidence against this hypothesis. Certainly, lead and calcium in bone have similar chemical properties and the substitution by lead for calcium in hydroxyapatite crystal [Ca_10_(PO_4_)_6_(OH)_2_] in bone tissue may result in a spurious increase in bone density when measured by DEXA because lead has a higher atomic number and attenuation coefficient than does calcium. This may in turn increase the attenuation of photons by the presence of lead in bone (Escribano et al. 1997; Popovic et al. 2004). However, a study has systematically evaluated this issue. Popovic et al. (2004) reported that DEXA overestimated BMD only by a slight 0.3% at a bone lead concentration of 100 ppm (1 ppm is here approximately equivalent to 1 μg/g), a very high bone lead level, or 0.0013 g/cm^2^/ppm lead. This small effect could not be detected by the Hologic QDR 4500A DEXA instrument, the device used for BMD measurement in the Popovic et al. (2004) study, which was also used in our study. We therefore believe that it is unlikely that our observation of a positive association between tibia lead and BMD arises from lead elevating measured BMD estimates.

A second explanation for this association is that lead may influence the function of bone cells. For example, lead may directly inhibit osteoblast function (Hass et al. 1967; Long et al. 1990a), cells responsible for bone formation (Marcus et al. 1996), or interfere with their ability to respond to hormonal regulation (e.g., by vitamin D_3_) (Pounds et al. 1991). Lead may also influence these cells indirectly by affecting circulating levels of PTH or vitamin D_3_ (Kristal-Boneh et al. 1998; Long et al. 1990a; Mason et al. 1990; Pounds et al. 1991). However, evaluation of the overall body of evidence supports the conclusion that the toxicity of lead in bone results in decreases in bone formation or increases in bone resorption. Therefore, higher lead levels in bone should be associated with lower BMDs, which is contrary to our findings. Thus, the toxicity of lead on bone cells is unlikely to explain our findings.

The third and final possible explanation that we considered, and the one we favor, is that higher BMD provides more sites for lead binding and deposition. That is, the two measures are positively associated because higher BMDs allow greater deposition of lead in bone, despite the fact that XRF results are normalized to bone mineral mass. Because lead deposits in bone tissue by substitution for calcium in the hydroxyapatite crystal during all stages of bone remodeling and bone growth (Castellino et al. 1995; Wittmers et al. 1988), higher amounts of calcium binding to the crystal in bone would result in both higher BMD measurements and more sites for lead deposition. For this explanation to be valid, we would expect that the same relationship would be observed in both men and women, but we only measured BMD in women, as previously explained. This explanation would also necessitate inequality between the “mineral” of BMD and XRF because the latter is normalized to photon scattering that occurs almost exclusively from bone mineral.

As expected, we found that *APOE* genotype was negatively associated with BMD. This finding is consistent with prior studies demonstrating that the *APOE* ε4 allele is associated with lower BMD and higher bone turnover rates (Shiraki et al. 1997; Zmuda et al. 1999). In addition, in the adjusted analysis, *APOE* genotype modified relations of tibia lead with BMD (with BMD as the dependent variable), and, similarly, BMD modified relations of *APOE* genotype with tibia lead (with tibia lead as the dependent variable). Taken together, these data imply that the ε4 allele was associated with lead loss that exceeded bone mineral loss in women with higher BMDs and that lead in bone does not lead to spurious elevations in BMD measurement (because if it does, we would expect the same relationship regardless of *APOE* allele).

Although we did not find that BMD modified the relations of tibia or patella lead with blood lead levels, we observed that BMD modified the relation of the Yale total energy index with blood lead levels. Higher total energy expenditure might be a risk factor for bone mineral loss in women with lower BMDs. Therefore, this may result in increased release of lead from bone back to blood in women with lower BMDs but may stabilize highly active bone such as patella from releasing lead back to blood in women with higher BMDs.

This study has several strengths. First, there was diversity by race/ethnicity and the inclusion of important genotypes, allowing us to evaluate relations of these important predictor variables with both lead biomarkers and BMD. Our sample size was > 50% larger than the only other study of the relation between tibia lead and BMD (Potula et al. 2006). Finally, ours was the first study, to our knowledge, to evaluate associations between BMD and patella lead levels. The limitations of the study include the cross-sectional design, which does not allow causal inferences. Second, the lead biomarkers were obtained from different visits (ranging from 18 to 36 months apart), and BMD was measured months after the measurements of other biomarkers. Therefore, the associations we found may not reflect actual cross-sectional associations of covariates and lead biomarkers, particularly for blood, BMD, and covariates that change over time. However, we do not think this problem is likely to be very severe for tibia and patella lead levels because residence time of lead in tibia is almost 3 decades (Rabinowitz et al. 1976) and in patella is 3–5 years (Chettle 1995; Todd and Chettle 1994). Third, we measured the lead biomarkers and BMD only one time, and mainly in women who had already passed menopause. The BMD and lead biomarker levels may have already significantly changed during this period, altering associations that may have existed during the premenopausal and perimenopausal periods. Finally, the various biomarkers and questionnaire-based variables were each measured with different measurement errors, and thus some of the contrasting associations could be explained by possible residual confounding.

We conclude that our data provide, to our knowledge, the first direct epidemiologic evidence that BMD may influence the deposition and kinetics of lead in bone. The findings suggest that the health effects of lead may be underestimated (but probably only slightly) for subjects with higher bone mineral densities. In addition, factors that alter bone turnover rates such as *APOE* and physical activity could have important influences on the kinetics of internal stores of lead.

## Figures and Tables

**Table 1:** Selected subject characteristics and variables by BMD group, Baltimore Memory Study, 2001–2005.

Variable	High BMD^a^ (*n* = 41)	Low BMD (*n* = 71)	No BMD* (*n* = 502)
Tibia lead (μg/g)	20.0 ± 13.8	19.6 ± 13.0	18.9 ± 12.9
Patella lead (μg/g)^b^	5.2 ± 15.5	6.0 ± 15.3	4.4 ± 19.4
Patella lead (μg/g)^c^	14.8 ± 8.7	14.9 ± 12.0	14.1 ± 11.4
Blood lead (μg/g)	3.2 ± 2.6	3.4 ± 1.9	2.9 ± 1.9
Age (years)	59.3 ± 6.1	59.9 ± 5.6	59.8 ± 5.9
Yale energy index (100 kcal/week)^d^,**	101.3 ± 69.4	71.8 ± 39.4	85.8 ± 63.2
Yale vigorous index^e^	18.0 ± 17.1	16.8 ± 15.6	14.3 ± 15.2
African American	17 (41.5)	30 (42.3)	215 (42.8)
Hormone replacement therapy**	24 (60.0)	26 (38.2)	178 (36.0)
Postmenopausal	35 (85.4)	60 (84.5)	436 (87.0)
*APOE* ε4 allele**	6 (14.6)	24 (33.8)	138 (27.7)

Values are mean ± SD or no. (%).

a *t*-Score > 0; *t*-score is the number of standard deviations above or below a young adult reference mean BMD.

b Included all subjects.

c Included only subjects who had patella lead level greater than zero (*n* = 69).

d Time spent for each activity on the Yale Physical Activity Survey is multiplied by an intensity (kcal/min) and is summed over all activities to create a total energy expenditure index for each subject.

e The frequency score is multiplied by the duration score to create the vigorous activity index (unitless).

* *p*-Values > 0.05, based on *t-*test statistics for continuous variables or from chi-square tests for binary and categorical variables, evaluating the statistical significance of the differences of mean covariates comparing females with and without BMD measurement.

** *p*-Values < 0.05, based on *t*-test statistics for continuous variables or from chi-square tests for binary and categorical variables, evaluating the statistical significance of the differences of mean covariates by BMD group.

**Table 2:** Linear regression modeling results identifying predictors of BMD, Baltimore Memory Study, 2001–2005.^a^

Independent variable (units of β coefficient)	β Coefficient	β SE^b^	*p*-Value
Model 1 (adjusted *r*^2^ = 29.0%)			
Intercept	0.664	0.073	< 0.01
Tibia (g/cm^2^/μg/g)	0.002	0.001	0.04
Weight (g/cm^2^/kg)	0.004	0.001	< 0.01
*APOE* ε4 allele (g/cm^2^)^c^	−0.057	0.029	0.05
Model 2 (adjusted *r*^2^ = 32.9%)^c^			
Intercept	0.610	0.078	< 0.01
Tibia (g/cm^2^/μg/g)	0.004	0.001	< 0.01
Weight (g/cm^2^/kg)	0.005	0.001	< 0.01
*ApoE* ε4 allele (g/cm^2^)^c^	−0.059	0.049	0.23
Tibia *ApoE (g/cm^2^/μg/g)	−0.005	0.002	< 0.01

a Models also controlled for the *VDR Fok*1 genotype.

b Robust estimates.

c *APOE* haplotype ε2 or ε3 versus ε4.

**Table 3:** Linear regression modeling results identifying predictors of blood lead levels, Baltimore Memory Study, 2001–2005.

Independent variable (units of β coefficient)	β Coefficient	β SE^a^	*p*-Value
Model 1 (adjusted *r*^2^ = 27.4%)^b^			
Intercept	2.247	0.538	< 0.01
Yale energy [ln(μg/dL)/100 kcal/week]	0.003	0.002	0.03
High BMD group^c^ [ln(μg/dL)] (vs. low)	0.481	0.237	0.05
High BMD group × Yale energy [ln(μg/dL)/100 kcal/week]	−0.006	0.003	0.02
Model 2 (adjusted *r*^2^ = 23.2%)^d^			
Intercept	2.654	0.450	< 0.01
Patella [ln(μg/dL)/μg/g]	0.010	0.005	0.03
High BMD group^c^ [ln(μg/dL)] (vs. low)	−0.013	0.147	0.93
High BMD group × patella [ln(μg/dL)/μg/g]	−0.005	0.009	0.57
Model 3 (adjusted *r*^2^ = 23.4%)^d^			
Intercept	2.490	0.437	< 0.01
Tibia [ln(μg/dL)/μg/g]	0.005	0.004	0.30
High BMD group^c^ [ln(μg/dL)] (vs. low)	−0.261	0.212	0.22
High BMD group × tibia [ln(μg/dL)/μg/g]	0.011	0.009	0.24

a Robust estimates.

b Model controlled for patella lead level (μg/g), BMI (kg/m^2^), consumption of alcohol in the past month (yes vs. no), HRT use (yes vs. no), and Yale vigorous index.

c *t*-Score > 0 versus *t*-score < 0; *t*-score is a number of SDs above or below a young adult reference mean BMD.

d Models also controlled for BMI (kg/m^2^), consumption of alcoholic beverages in the past month (yes vs. no), hormone replacement use (yes vs. no), and the Yale vigorous activity index.

**Table 4:** TOBIT regression modeling results identifying predictors of tibia lead levels,^a^ Baltimore Memory Study, 2001–2005.

Independent variable (units of β coefficient)	β coefficient	β SE^b^	*p*-Value
Model 1 (adjusted *r*^2^ = 16.2%)			
Intercept	8.677	7.822	0.27
Age (μg/g/year)	0.471	0.209	0.02
African American (μg/g)	8.756	2.795	< 0.01
BMD (μg/g per mg/cm^2^)	0.013	0.007	0.04
Model 2 (adjusted *r*^2^ = 19.4%)^c^			
Intercept	18.929	4.667	< 0.01
*ApoE* ε4 allele (μg/g)^d^	6.163	3.314	0.06
High BMD group^e^ (μg/g)	4.294	2.729	0.12
High BMD group *ApoE ε4 allele (μg/g)	−16.077	4.340	< 0.01
Model 3 (adjusted *r*^2^ = 18.1 %)^c^			
Intercept	14.934	5.401	< 0.01
Yale energy (μg/g/100 kcal/wk)	0.088	0.036	0.02
High BMD group^e^ (μg/g)	8.090	4.581	0.08
High BMD group × Yale energy (μg/g/100 kcal/wk)	−0.098	0.045	0.03

a Models controlled for education, alcohol use in the past month (yes vs. no), and dietary vitamin C intake.

b Robust estimates.

c Models also included age and race/ethnicity.

d At least one *APOE* ε4 allele vs. none.

e *t*-Score > 0 vs. *t*-score < 0 ; *t*-score is a number of SDs above or below a young adult reference mean BMD.
